# *Notes from the Field:* Cluster of Acute Flaccid Myelitis in Five Pediatric Patients — Maricopa County, Arizona, 2016

**DOI:** 10.15585/mmwr.mm6628a4

**Published:** 2017-07-21

**Authors:** Sally A. Iverson, Scott Ostdiek, Siru Prasai, David M. Engelthaler, Melissa Kretschmer, Nicole Fowle, Harlori K. Tokhie, Janell Routh, James Sejvar, Tracy Ayers, Jolene Bowers, Shane Brady, Shannon Rogers, W. Allan Nix, Ken Komatsu, Rebecca Sunenshine, AFM Investigation Team, AFM Investigation Team, Tammy Sylvester, Veronica Harrison, Jennifer Heim, Susan Robinson, Gholamabbas A. Ostovar, Kathryn Fitzpatrick

**Affiliations:** ^1^Epidemic Intelligence Service, CDC; ^2^Maricopa County Department of Public Health, Phoenix, Arizona; ^3^Arizona Department of Health Services; ^4^Phoenix Children’s Hospital, Arizona; ^5^Translational Genomics Research Institute, Flagstaff, Arizona; ^6^Division of Viral Diseases, National Center for Immunization and Respiratory Diseases, CDC; ^7^Division of High-Consequence Pathogens and Pathology, National Center for Emerging and Zoonotic Infectious Diseases, CDC; ^8^Career Epidemiology Field Officer Program, CDC; ^9^Maricopa Integrated Health System, Phoenix, Arizona.; Maricopa County Department of Public Health, Arizona; Translational Genomics Research Institute, Flagstaff, Arizona; Phoenix Children’s Hospital, Arizona; Arizona Department of Health Services; Maricopa Integrated Health System, Arizona; Arizona State Public Health Laboratory, Virology Section.

In 2016, CDC saw an increase in cases of acute flaccid myelitis (AFM); 144 persons in 37 states and the District of Columbia were confirmed to have AFM. After investigations in California (*1*) and Colorado (*2*) in 2014, CDC characterized AFM as an acute flaccid paralysis (AFP) distinguishable by magnetic resonance imaging (MRI) abnormalities of the gray matter of the anterior and posterior spinal cord segments, involving one or more spinal segments (*3*). Although certain viruses (e.g., nonpoliovirus enteroviruses, adenoviruses, and West Nile virus) can cause rare cases of AFP, and findings from the 2014 outbreak investigations indicated that enterovirus D68 (EV-D68) was temporally associated with AFM, no viral etiology for AFM has been definitively established (*3*). In September 2016, an acute care hospital in Arizona notified the Maricopa County Department of Public Health (MCDPH) of a suspected case of AFM and subsequent cluster of 11 children who were evaluated with similar neurologic deficits; differential diagnoses included transverse myelitis and AFM. The Maricopa County Department of Public Health, in cooperation with the Arizona Department of Health Services, CDC, the Translational Genomics Research Institute (TGen, Flagstaff, Arizona), and the acute care hospital, initiated an investigation to confirm AFM cases and identify an etiology.

The 2015 Council of State and Territorial Epidemiologists and CDC case definition for probable AFM requires acute onset of flaccid limb weakness and cerebrospinal fluid (CSF) pleocytosis (CSF white blood cell [WBC] count >5/mm^3^ when corrected for red blood cells). A confirmed case must have an MRI demonstrating lesions restricted primarily to the gray matter of the spinal cord, in addition to acute onset of flaccid limb weakness (*4*). Based on medical chart abstraction and review of the MRI images, a CDC neurology subject matter expert verified four confirmed cases of AFM and one probable case. Among the six patients whose cases did not meet the AFM confirmed or probable case definition, two had focal limb weakness and pleocytosis (CSF WBC = 7/mm^3^ and 22/mm^3^, respectively), but MRI results indicated alternative etiologies (acute disseminated encephalomyelitis and neuromyelitis optica, respectively). The case that met the probable case definition had pleocytosis (CSF WBC = 7/mm^3^), but MRI findings were inconsistent with AFM, and no other plausible diagnosis was identified.

Onset dates for the four confirmed cases occurred during August 19–September 15, 2016. All four patients had preceding respiratory (three patients) or gastrointestinal illness (one patient), with onset dates for those illnesses occurring during August 14–September 13. Their illness began a median of 2 days (range = 2–5 days) before onset of focal limb weakness; three patients experienced tactile or measured fever preceding onset of neurologic symptoms (Table). Among patients with confirmed cases, focal limb weakness was present in a single limb (one case), three limbs (two cases), and four limbs (one case). Two patients with confirmed cases and one patient with a probable case had a prior medical history of asthma, and a third patient with a confirmed case reported a family history of asthma. The investigation team conducted hypothesis-generating interviews with all confirmed AFM patients and their proxies. Three of the four patients with confirmed cases were residents of Maricopa County, and no epidemiologic links were detected among the four patients. None of the patients had traveled to an area with ongoing Zika virus transmission in the month prior to symptom onset.

**TABLE:** Characteristics of five pediatric patients with acute flaccid myelitis* — Maricopa County, Arizona, October 2016

Characteristic	Patient 1	Patient 2	Patient 3	Patient 4	Patient 5
Case status	Confirmed	Confirmed	Confirmed	Confirmed	Probable
Age at onset (yrs)	3.5	10	4	9	12
Sex	Boy	Girl	Girl	Girl	Girl
Onset of focal limb weakness	August 23, 2016	August 19, 2016	September 15, 2016	September 8, 2016	August 27, 2016
Onset of preceding respiratory or gastrointestinal illness	August 21, 2016 (respiratory)	August 14, 2016 (respiratory)	September 13, 2016 (respiratory)	September 6, 2016 (gastrointestinal)	August 17, 2016 (respiratory)
Presence of fever (tactile^†^ or measured)	Yes	No	Yes	Yes	No
Limbs affected (region)	1 (LUE)	4	3 (BUE, RLE)	4	1 (LUE)
Cranial nerve features and timing	None	Facial droop subsequent to onset of limb weakness	Facial droop subsequent to onset of limb weakness	Facial droop before onset of limb weakness	Diplopia concurrent with limb weakness
Patient and family history of asthma	Asthma and family history of asthma	Asthma	None	Family history of asthma	Mild asthma, seasonal allergies, food allergies, eczema
Corticosteroid history	Maintenance inhaled fluticasone; oral budesonide for asthma exacerbation August 15–19	Maintenance inhaled fluticasone; oral prednisolone for asthma exacerbation beginning August 14	None	Oral prednisolone for treatment of Bell’s palsy beginning September 5	Maintenance inhaled fluticasone
Magnetic resonance imaging (MRI) findings	T2 signal abnormalities in anterior and posterior columns of the central gray cervical cord	T2 signal abnormality with anterior and posterior involvement, contiguous through multiple levels of the cord	T2 signal abnormality in the anterior horn of the central gray cord	Anterior horn signal abnormality extending four cervical levels	Normal MRI result
Cerebrospinal fluid/white blood cell/mm^3^	50	150	207	115	7
Nasopharyngeal swab polymerase chain reaction results from TGen	Positive for EV-D68	Positive for EV-D68	Positive for EV-D68	Unavailable	Unavailable
Stool specimen testing results	Negative enterovirus/parechovirus by RT-PCR	Negative viral culture and enterovirus/parechovirus by RT-PCR	Negative viral culture and enterovirus/parechovirus by RT-PCR	Positive for coxsackievirus A10 by Sanger sequencing of the VP1 region	Unavailable

**Abbreviations**: BUE = bilateral upper extremities; EV = enterovirus; LUE = left upper extremity; RLE = right lower extremity; RT-PCR = reverse transcription–polymerase chain reaction; T2 = T2 weighted image; TGen = Translational Genomics Research Institute (Flagstaff, Arizona); VP1 = viral protein 1.

* Four with confirmed cases and one with a probable case.

^†^ Felt warm to the touch, according to parent.

CSF was collected from all four patients with confirmed AFM. Median CSF WBC count was 133/mm^3^ (range = 50–207), and initial viral testing at the hospital included CSF reverse transcription–polymerase chain reaction (RT-PCR) assays for enterovirus (three patients) and West Nile virus (WNV) (two patients), polymerase chain reaction (PCR) assay for herpes simplex virus (two patients), and enzyme immunoassay to detect immunoglobulin M (IgM) or immunoglobulin G (IgG) for WNV (three patients). All results were negative. All four CSF specimens were negative on TGen amplicon sequencing assay (*5*,*6*) using primers based on the 2014 circulating EV-D68 virus. Results of microbiome analysis by metagenomic sequencing of RNA and 16S rRNA gene sequencing of DNA extracted from CSF revealed bacterial sequencing dominated by *Propionibacterium*, which is a normal component of the skin flora and most likely represents a contaminant (*7*). Serum collected from one patient at initial evaluation was negative for WNV IgM and IgG on a hospital immunoassay; serum collected from the same patient 47 days after onset of focal limb weakness and from two additional patients (19 and 26 days after onset of focal limb weakness) were negative for WNV IgM and St. Louis encephalitis IgM at the Arizona State Public Health Laboratory.

Three of the four patients had nasopharyngeal (NP) swabs available from initial evaluation that were forwarded to CDC; one specimen was positive for enterovirus/rhinovirus on a panviral respiratory PCR panel at the admitting hospital laboratory and for EV-D68 at CDC. RNA extracted from NP swabs from all three patients was positive by the TGen amplicon sequencing test for EV-D68 (GenBank Bioproject); an NP specimen from a patient who did not meet the AFM confirmed or probable case definitions also was positive for EV-D68 by the same assay.

Stool specimens were collected from two patients at the time of initial evaluation; vital cultures of these specimens were negative on viral. One available specimen and three additional specimens, collected 28, 47, and 63 days after onset of focal limb weakness, were sent to CDC for four enterovirus/parechovirus RT-PCR assays. A stool specimen, collected at day 28 from the patient who did not have an NP swab available, was positive for coxsackievirus A10.

This cluster of AFM at one children’s acute care hospital is the largest cluster identified to date in Arizona and is part of a nationally identified increase in AFM cases. Although no statewide surveillance system specific to AFM is available, this cluster was detected by physician reporting, highlighting the need for physicians to remain vigilant for this emerging disease and to report cases that fit the AFM case definition to their local health department. Metagenomic analyses identified EV-D68 in NP swabs from the three patients for whom specimens were available, along with a specimen from a patient who did not meet the AFM case definition; therefore, no single etiology or risk factor was associated with only confirmed cases. 

Patient and family history of asthma was the most common comorbidity reported among confirmed AFM cases and should be considered in future case investigations. Expanded analysis of infectious, postinfectious, and noninfectious etiologies might provide further insight into the mechanism of AFM.

## References

[1] CDC. Notes from the field: acute flaccid myelitis among persons aged ≤21 years—United States, August 1–November 13, 2014. MMWR Morb Mortal Wkly Rep 2015;63:1243–4. 25577990 PMC4646045

[2] Pastula DM, Aliabadi N, Haynes AK, Acute neurologic illness of unknown etiology in children—Colorado, August–September 2014. MMWR Morb Mortal Wkly Rep 2014;63:901–2. 25299607 PMC4584613

[3] Sejvar JJ, Lopez AS, Cortese MM, Acute flaccid myelitis in the United States, August–December 2014: results of nationwide surveillance. Clin Infect Dis 2016;63:737–45. 10.1093/cid/ciw372 27318332 PMC5709818

[4] Council of State and Territorial Epidemiologists (CSTE). CSTE position statement: standardized case definition for acute flaccid myelitis. Atlanta, GA: Council of State and Territorial Epidemiologists; 2015. http://c.ymcdn.com/sites/www.cste.org/resource/resmgr/2015PS/2015PSFinal/15-ID-01.pdf

[5] Colman RE, Anderson J, Lemmer D, Rapid drug susceptibility testing of drug-resistant *Mycobacterium tuberculosis* isolates directly from clinical samples by use of amplicon sequencing: a proof-of-concept study. J Clin Microbiol 2016;54:2058–67. 10.1128/JCM.00535-16 27225403 PMC4963505

[6] Bowers JR, Lemmer D, Sahl JW, KlebSeq, a diagnostic tool for surveillance, detection, and monitoring of *Klebsiella pneumoniae*. J Clin Microbiol 2016;54:2582–96. 10.1128/JCM.00927-16 27510832 PMC5035412

[7] Grice EA, Segre JA. The skin microbiome. Nat Rev Microbiol 2011;9:244–53. 21407241 10.1038/nrmicro2537 PMC3535073

